# Genome Sequences of Two Pseudorabies Virus Strains Isolated in Greece

**DOI:** 10.1128/genomeA.01624-15

**Published:** 2016-01-21

**Authors:** Konstantinos V. Papageorgiou, Nicolás M. Suárez, Gavin S. Wilkie, George Filioussis, Nikolaos Papaioannou, Hans J. Nauwynck, Andrew J. Davison, Spyridon K. Kritas

**Affiliations:** a MRC–University of Glasgow Centre for Virus Research, Glasgow, United Kingdom; b Department of Microbiology and Infectious Diseases, Veterinary Faculty, School of Health Sciences, Aristotle University of Thessaloniki, Thessaloniki, Greece; c Department of Pathology, Veterinary Faculty, School of Health Sciences, Aristotle University of Thessaloniki, Thessaloniki, Greece; d Laboratory of Virology, Faculty of Veterinary Medicine, Ghent University, Ghent, Belgium

## Abstract

Pseudorabies virus (species *Suid herpesvirus 1*) belongs to the genus *Varicellovirus*, subfamily *Alphaherpesvirinae*, family *Herpesviridae*, and is the causative agent of an acute and frequently fatal disease that affects mainly pigs. Here, we report the genome sequences of two strains of this virus isolated in Greece in 2010.

## GENOME ANNOUNCEMENT

Pseudorabies virus (PRV) is the causative agent of Aujeszky’s disease, an acute and frequently fatal disease that affects pigs and, incidentally, certain other domestic and wild animals (1). As a part of an epidemiological study conducted in Greece from 2008 to 2013, two PRV strains were isolated in 2010 from farms in the northern part of the country located approximately 100 km apart. We determined the genome sequences of these strains, which were named Kolchis and Hercules.

The strains were isolated from brain tissue specimens from two newborn piglets showing the typical neurological signs of Aujeszky’s disease by passaging twice in swine testicle cells (ST) (ATCC CRL-1746). DNA was extracted from the medium of the final passage by using a DNeasy blood and tissue kit (Qiagen). An aliquot of DNA from each sample (66.5 and 60 ng for strains Kolchis and Hercules, respectively) was sheared acoustically to a modal size of 550 bp using a Covaris S220 sonicator (Covaris, Inc.). The sheared DNA fragments were prepared for sequencing by using a Kapa library preparation kit (Kapa Biosystems), as described elsewhere (2). A MiSeq platform (Illumina) was used to generate 7,033,866 and 4,661,406 300-base paired-end reads for strains Kolchis and Hercules, respectively. Poor-quality reads were removed using Trim Galore version 0.2.2 (http://www.bioinformatics.babraham.ac.uk/projects/trim_galore), and reads from the pig genome (GenBank accession no. GCA_000003025.4) were removed by using BWA version 0.6.2-r126 (3). The remaining reads (3,842,770 reads for strain Kolchis, and 2,031,656 reads for strain Hercules) were assembled *de novo* using SPAdes 3.5.0 (4). scaffold_builder (5) was used to produce a draft genome sequence by scaffolding the resulting contigs against the sequence of strain Kaplan (GenBank accession no. JQ809328.1 [6]), and the remaining gaps were closed by using GapFiller version 1-11 (7). The integrity of the final sequences was verified by aligning them against the trimmed read data using BWA and visualizing the alignments in Tablet version 1.13.08.05 (8). For strain Kolchis, 2,845,302 reads aligned at an average coverage of 3,440 reads per nucleotide, and for strain Hercules, these values were 1,233,865 reads and 1,522 reads, respectively.

The complete strain Kolchis genome is 141,542 bp in size and has a G+C content of 73.7%. The long unique (U_L_) and short unique (U_S_) regions are 100,497 and 8,761 bp in size, respectively, and the terminal repeats (TR_S_) and inverted repeats (IR_S_) flanking U_S_ are 16,142 bp in size. Strains Kolchis and Hercules differ by only 10 nucleotide substitutions (not including TR_S_), whereas the American strain Kaplan is more divergent, differing from strain Kolchis by 663 substitutions; these values do not take inserted or deleted nucleotides into account. Like other PRV isolates, strains Kolchis and Hercules contain 69 open reading frames predicted to encode functional proteins (9).

To our knowledge, these are the first reported genome sequences of PRV strains isolated in Greece. They will aid work on the diagnosis and epidemiology of Aujeszky’s disease, which is widespread in this region.

### Nucleotide sequence accession numbers. 

The PRV strain Kolchis and Hercules genome sequences have been deposited in GenBank under the accession numbers KT983811 and KT983810, respectively.
